# Prevention of β-amyloid degeneration of microglia by erythropoietin depends on Wnt1, the PI 3-K/mTOR pathway, Bad, and Bcl-xL

**DOI:** 10.18632/aging.100440

**Published:** 2012-03-03

**Authors:** Yan Chen Shang, Zhao Zhong Chong, Shaohui Wang, Kenneth Maiese

**Affiliations:** ^1^ Laboratory of Cellular and Molecular Signaling; ^2^ Cancer Institute of New Jersey; ^3^ New Jersey Health Sciences University, Newark, New Jersey 07101

**Keywords:** Alzheimer's disease, amyloid, erythropoietin, microglia, mTOR, Wnt

## Abstract

Central nervous system microglia promote neuronal regeneration and sequester toxic β-amyloid (Aβ) deposition during Alzheimer's disease. We show that the cytokine erythropoietin (EPO) decreases the toxic effect of Aβ on microgliain vitro. EPO up-regulates the cysteine-rich glycosylated wingless protein Wnt1 and activates the PI 3-K/Akt1/mTOR/ p70S6K pathway. This in turn increases phosphorylation and cytosol trafficking of Bad, reduces the Bad/Bcl-x_L_ complex and increases the Bcl-x_L_/Bax complex, thus preventing caspase 1 and caspase 3 activation and apoptosis. Our data may foster development of novel strategies to use cytoprotectants such as EPO for Alzheimer's disease and other degenerative disorders.

## INTRODUCTION

More than twenty-five million individuals may suffer from Alzheimer's disease, pre-senile dementia, and other disorders of cognitive loss throughout the world [1, 2]. β-amyloid (Aβ) deposition in the brain is considered to be a significant component of Alzheimer's disease that leads to progressive neuro-degeneration. Aβ toxicity can involve multiple cell types including neurons [3-6], vascular cells [2, 7, 8], and inflammatory microglial cells [7, 9]. In regard to microglia, these inflammatory cells can form a protective network for the brain to control neurotrophic factors [10], limit oxidative stress [11, 12], and foster neuronal regeneration [13]. Microglia also may limit the deposition and toxicity of Aβ and promote neuronal survival [4, 9, 14-16].

In light of the significance microglia may hold as therapeutic targets directed against Alzheimer's disease, it becomes vital to understand the cellular pathways that maintain microglia survival during Aβ toxicity. One therapeutic agent that may provide protection against both Alzheimer's disease and microglial injury is the cytokine and growth factor erythropoietin (EPO) [3, 17-19]. EPO has broad protective effects in the heart, the vasculature, and the nervous system [20-28]. EPO also is involved in immune system modulation [29-31] and controls the activation and proliferation of microglia [24, 32-35]. Recent studies have suggested that the wingless cysteine-rich glycosylated protein Wnt1 [36-38] may limit the production and toxicity of Aβ[39, 40]. Wnt1 and the wingless pathway offer cellular protection through non-neuronal cells including microglia [4, 41-43] and mediate EPO cytoprotection in experimental models of diabetes mellitus and hypoxia [20, 44, 45]. Interestingly, Wnt1 does not entirely rely upon traditional canonical and non-canonical pathways [46, 47] and can function through the phosphoinositide 3-kinase (PI 3-K) and Akt1 pathways in the apoptotic cascade [4, 20, 41, 43, 48].

Given that Wnt1 can prevent apoptosis through PI 3-K mediated pathways [49-51], we investigated whether EPO and Wnt1 employed the PI 3-K/Akt1/mammalian target of rapamycin (mTOR) pathways to protect microglia during Aβ toxicity. We show that Wnt1 is a central component for EPO to promote microglial integrity and prevent the loss of these cells during early and late apoptotic injury with Aβ exposure. Wnt1 depends upon PI 3-K, Akt1, mTOR, and p70S6K to maintain microglial viability during Aβ exposure. Wnt1 regulates the apoptotic cascade by maintaining mitochondrial membrane potential, phosphorylating and fostering the translocation of Bad from the mitochondria to the cytosol, reducing the Bad/Bcl-x_L_ complex, increasing the Bcl-x_L_/Bax complex, and blocking caspase 1 and caspase 3 activation throughBcl-x_L_.

## RESULTS

### EPO protects microglia against beta-amyloid _1-42_ (Aβ) exposure through Wnt1

**Figure 1:**
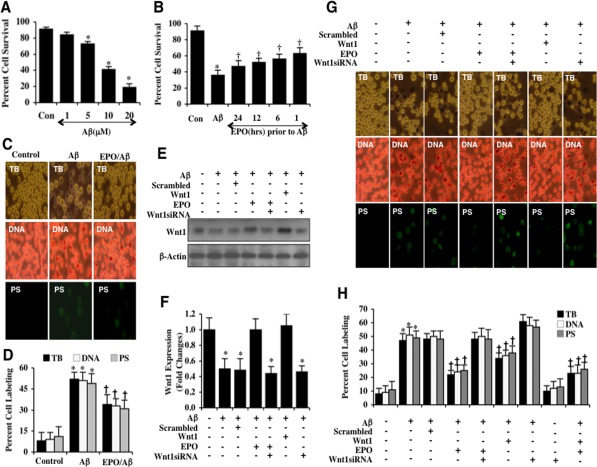
EPO preserves microglia survival against Aβ through Wnt1 (**A**) Microglia were exposed to Aβ(10 μM) at the concentrations of 1, 5, 10, and 20 μM and cell survival was determined 24 hours after administration of Aβwith trypan blue dye exclusion method (*P< 0.01 vs. Control). Con = control = untreated microglia. Each data point represents the mean and SEM from 6 experiments. **(B)** EPO (10 ng/ml) was applied to microglial cultures at 24, 12, 6, or 1 hour prior to administration of Aβ (10 μM) and cell survival was determined 24 hours after Aβ administration with the trypan blue dye exclusion method (*P<0.01 vs. untreated control; ^†^*P* <0.05 *vs*. Aβ). Each data point represents the mean and SEM from 6 experiments. Con = control = untreated microglia. **(C** and **D)** EPO (10 ng/ml) was applied to microglial cultures 1 hour prior to the administration of Aβ (10 μM) and cell survival, DNA fragmentation, and PS exposure were determined 24 hours later. Representative images **(C)** and quantitative analysis **(D)** demonstrate that Aβ leads to a significant increase in trypan blue staining, DNA fragmentation, and membrane PS exposure in microglia 24 hours after Aβ exposure compared to untreated control cultures. EPO (10 ng/ml) applied 1 hour prior to Aβ exposure prevented microglial cell injury, DNA fragmentation, and membrane PS exposure(*P < 0.01 vs. Control; ^†^*P* <0.05 *vs*. Aβ). Each data point represents the mean and SEM from 6 experiments. **(E** and **F)** EPO (10 ng/ml) or Wnt1 (100 ng/ml) were applied 1 hour prior to Aβ (10 μM) administration with Wnt1 expression determined 6 hours following Aβ exposure. EPO (10 ng/ml) and Wnt1 (100 ng/ml) maintained the expression of Wnt1 that is otherwise down-regulated during Aβ exposure. Gene reduction of*Wnt1*with Wnt1 siRNA significantly reduced the expression of Wnt1 following a 6 hour period of Aβ exposure or treatment with EPO (10 ng/ml) during Aβ exposure. Non-specific scrambled siRNA did not alter Wnt1 expression during Aβ exposure (*P<0.01 vs. Control). (**G** and **H**) EPO was applied to microglial cultures 1 hour prior to the administration of Aβ and trypan blue dye exclusion, DNA fragmentation, and membrane PS exposure were determined 24 hours later. Representative images (**G**) and quantitative results (**H**) show that EPO (10 ng/ml) or Wnt1 (100 ng/ml) applied 1 hour prior to Aβ significantly reducedtrypan blue staining, DNA fragmentation, and membrane PS exposure in microglia 24 hours after Aβ exposure. Gene reduction of *Wnt1* with transfection of Wnt1 siRNA prior to Aβ exposure prevented EPO (10 ng/ml) from blocking cell injury and resulted in increased trypan blue staining, DNA fragmentation, and membrane PS exposure in microglia 24 hours following Aβ exposure. Non-specific scrambled siRNA did not significantly alter microglial cell injury following Aβ exposure (*P<0.01 vs. untreated control; ^†^*P* < 0.05 *vs*. Aβ). Each data point represents the mean and SEM from 6 experiments.

Aβ was applied to microglia in a series of concentration (1, 5, 10, and 20 μM) and microglial cell survival was assessed 24 hours later by the trypan blue dye exclusion method. As shown in Figure 1A, Aβ significantly decreased cell survival to 73 ± 3%, 41 ± 3%, and 19 ± 4% at the concentrations of 5 μM, 10 μM, and 20 μM respectively 24 hours following Aβ administration when compared to untreated control cells with a survival of 92 ± 6%. The Aβ concentration of 1 μM was not significantly toxic with a cell survival of 84 ± 7%. Since the Aβ concentration of 10 μM resulted in a survival of approximately 40% (60% microglial cell loss), this concentration of Aβ was employed for the subsequent experimental paradigms.

We investigated the ability of EPO to prevent microglial cell injury following Aβ exposure. A concentration of EPO 10 ng/ml was used since this concentration has previously been shown to provide significant cytoprotection without toxicity in several experimental systems including those involving Aβ [20, 21]. EPO (10 ng/ml) was applied to microglia at 24, 12, 6, and 1 hour prior to the administration of Aβ and cell survival was determined 24 hours later. As shown in Figure 1B, cell survival was increased from 36 ± 6% in cells exposed to only Aβ alone to 63 ± 7% (1 hour), 58 ± 6% (6 hours), 55 ± 4% (12 hours), and 47 ± 7% (24 hours) respectively, illustrating that administration of EPO closest to the point of Aβ exposure yielded the greatest degree of cytoprotection for microglia. We therefore utilized a 1 hour application of EPO prior to Aβ exposure for subsequent studies.

In Figures 1C and 1D, trypan blue dye exclusion staining was used to assess microglial cell injury. Early apoptotic injury was assessed by membrane phosphatidylserine (PS) exposure (annexin V staining) and late apoptotic genomic DNA fragmentation was assessed by TUNEL 24 hours following Aβ exposure. As shown in Figures 1C and 1D, representative images and quantitative results demonstrate that Aβ led to a significant increase in trypan blue staining, DNA fragmentation, and membrane PS exposure in microglia at 24 hours after Aβ exposure when compared to untreated control cultures. Yet, treatment with EPO (10 ng/ml) 1 hour prior to Aβ exposure significantly decreased trypan blue dye uptake, DNA fragmentation, and membrane PS exposure in microglia 24 hours following Aβ administration (Figures 1C and 1D).

We examined the ability of EPO to alter the expression of Wnt1 in microglia during Aβ exposure. Western blot assay was performed for the endogenous cellular expression of Wnt1 at 6 hours following Aβ administration. A representative Western blot demonstrates that Wnt1 expression was decreased within 6 hours following Aβ exposure (Figures 1E and 1F). Application of EPO (10 ng/ml) in microglia significantly maintained the expression of Wnt1 at 6 hours after Aβ exposure (Figures 1E and 1F), illustrating that EPO can maintain the expression of Wnt1 during Aβ exposure.

As shown in Figure 1E and 1F, transfection with Wnt1 siRNA in microglia resulted in the significant reduction of Wnt1 expression as revealed by Western blot analysis at 6 hours following Aβ exposure. As a control, non-specific scrambled siRNA did not alter Wnt1 protein expression in untreated control microglia or in microglia exposed to Aβ alone, demonstrating that Wnt1 siRNA was specific to block protein expression of Wnt1. Application of EPO (10 ng/ml) or of Wnt1 (100 ng/ml) 1 hour prior to Aβ exposure significantly maintained the expression of Wnt1 similar to control cultures. However, gene reduction of *Wnt1* with siRNA in the presence of EPO (10 ng/ml) prevented EPO from maintaining the expression of Wnt1 during Aβ exposure (Figures 1E and 1F).

We investigated whether gene reduction of *Wnt1* could impact protection by EPO. As shown in Figure 1G, representative images demonstrate that Aβ exposure leads to a significant increase in trypan blue staining, genomic DNA fragmentation, and PS membrane externalization in microglia 24 hours later. EPO (10 ng/ml) or Wnt1 (100 ng/ml) prevented cell injury, DNA fragmentation, and membrane PS exposure (Figures 1G and 1H). In contrast, gene reduction of *Wnt1* with siRNA increased cell injury when compared with Aβ alone, suggesting that endogenous Wnt1 protein is also necessary for microglial protection during Aβ exposure (Figures 1G and 1H). In addition, gene reduction of *Wnt1* with siRNA during EPO application significantly blocked protection by EPO, also illustrating that Wnt1 is required for EPO cytoprotection in microglia during Aβ exposure (Figures 1G and 1H). Interestingly combined treatment with EPO (10 ng/ml) and Wnt1 (100 ng/ml) provides similar protection when compared with EPO alone treatment and slightly improved protection when compared with Wnt1 treatment alone, suggesting that EPO relies on Wnt1 for cellular protection against Aβ exposure, but also may utilize other signal transduction pathways (Figure 1H).

### EPO maintains the activity of Akt1 through Wnt1 during Aβ exposure

**Figure 2:**
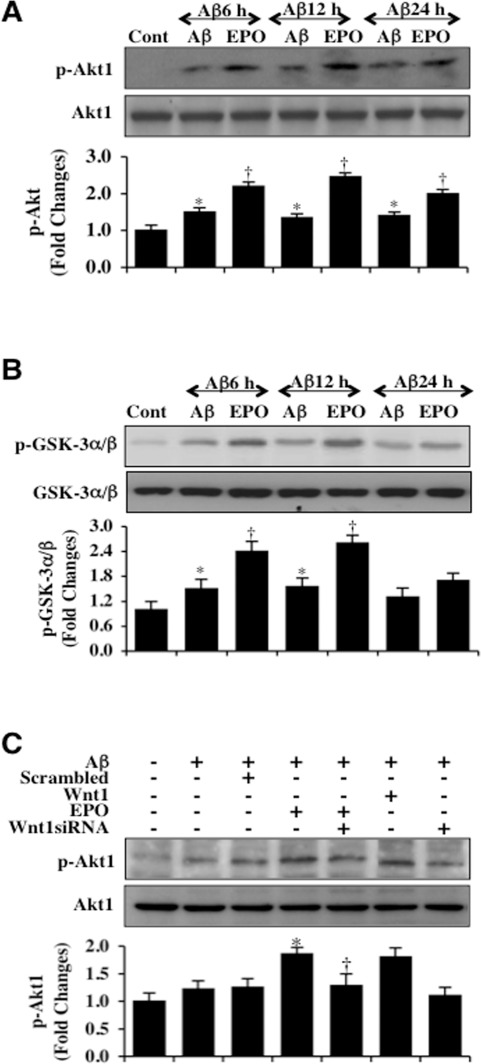
EPO maintains Akt1 activation through Wnt1 during Aβ exposure **(A)** Microglial protein extracts (50 μg/lane) were immunoblotted with phosphorylated Akt1 (p-Akt1) (active form) at 6, 12, and 24 hours following administration of Aβ (10 μM). Aβ resulted a mild increase in the expression of p-Akt1 over a 24 hour period. EPO (10 ng/ml) with a 1 hour pretreatment significantly increased the expression of p-Akt1 over a 24 hour period following Aβ exposure (*P < 0.01 vs. Control; ^†^*P*< 0.01 vs. Aβ of corresponding time point). In all cases, each data point represents the mean and SEM from 3 experiments. (**B**) Akt1 activity in microglia was determined with a GSK-3β fusion protein through assessment of p-GSK-3α/β expression following Aβ exposure. EPO (10 ng/ml) with a 1 hour pretreatment significantly increased the activity of Akt1 over 12 hours following Aβ exposure (*P <0.01 vs. Control; ^†^*P*<0.01 vs. Aβ of corresponding time point). In all cases, each data point represents the mean and SEM from 3 experiments. (**C**) Gene reduction of Wnt1 was performed with transfection of Wnt1 siRNA prior to Aβ exposure in microglia and p-Akt1 expression was determined at 6 hours following Aβ exposure. Loss of Wnt1 resulted in a decreased expression of p-Akt1 following a 6 hour period of Aβ exposure and significantly reduced EPO (10 ng/ml) expression of p-Akt1 during Aβ exposure. Non-specific scrambled siRNA did not alter p-Akt1 expression during Aβ exposure (*P < 0.01 vs. Aβ; ^†^*P* < 0.01 vs. EPO/Aβ).

Western blot assay for the expression of phosphorylated Akt1 (p-Akt1) (activated form of Akt1) was performed following Aβ exposure. As shown in Figure 2A, the expression of p-Akt1 was mildly increased at 6, 12, and 24 hours following Aβ exposure. Application of EPO (10 ng/ml) 1 hour prior to Aβ significantly increased the expression of p-Akt1 6 hours following Aβ and maintained the increased expression of p-Akt1 over a 24 hour following Aβ administration (Figure 2A). In a similar manner, we assessed Akt1 activity through a GSK-3β fusion protein (Figure 2B). EPO administration 1 hour prior to Aβ also significantly increased and maintained the activity of Akt1 determined by the expression of p-GSK-α/β when compared to microglia exposed to Aβ only (Figure 2B).

We assessed the ability of EPO to maintain activation of Akt1 during gene reduction of Wnt1. As shown in Figure 2B, representative images demonstrate that EPO (10 ng/ml) or Wnt1 (100 ng/ml) significantly increase the expression of p-Akt1 at 6 hours following Aβ exposure. However, gene reduction of *Wnt1* with siRNA significantly during EPO application significantly reduced the expression of p-Akt1 at 6 hours following Aβ exposure in microglia (Figure 2C), illustrating that Wnt1 is a necessary component for EPO to maintain the activity of Akt1 during Aβ exposure.

### EPO requires Wnt1, mTOR and p70S6K and the PI 3-K/Akt1 pathway to protect microglia during Aβ exposure

**Figure 3:**
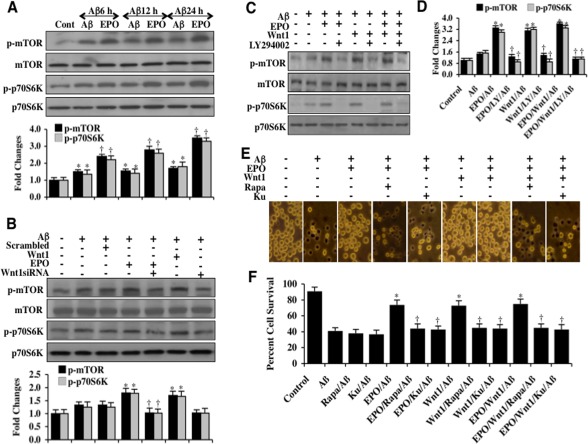
EPO oversees Wnt1, mTOR and p70S6K and the PI 3-K/Akt1 pathway to protect microglia during Aβ exposure **(A)** Microglial protein extracts (50 μg/lane) were immunoblotted with phosphorylated mTOR (p-mTOR, (Ser^2448^)) and phosphorylated p70S6K (p-p70S6K, Thr^389^)) antibodies at 6, 12, and 24 hours following exposure to Aβ (10 μM). Aβ resulted in a slight increase in the expression of p-mTOR and p-p70S6K. In contrast, EPO (10 ng/ml) with a 1 hour pretreatment significantly increased the expression of p-mTOR and p-p70S6K during Aβ exposure (*P <0.01 vs. Control; †*P*<0.01 vs. Aβ of corresponding exposure time). In all cases, each data point represents the mean and SEM from 3 experiments. (**B)** Gene reduction of*Wnt1*was performed with transfection of Wnt1 siRNA prior to Aβ (10 μM) exposure in microglia. Expression of p-mTOR and p-p70S6K were determined at 6 hours following Aβ exposure. EPO (10 ng/ml) or Wnt1 (100 ng/ml) applied 1 hour prior to Aβ exposure significantly increased the expression of p-mTOR and p-p70S6K. Transfection with Wnt1 siRNA significantly reduced the expression of p-mTOR and p-p70S6K following a 6 hour period of Aβ exposure and during EPO (10 ng/ml) administration with Aβ exposure. Non-specific scrambled siRNA did not alter the expression of p-mTOR and p-p70S6K during Aβ exposure (*P<0.01 vs. Aβ; ^†^*P*<0.01 vs. EPO/Aβ). **(C** and **D)** EPO (10 ng/ml), Wnt1 (100 ng/ml), or combined EPO with Wnt1 were applied to microglial cultures 1 hour prior to Aβ(10 μM) exposure and p-mTOR and p-p70S6K expression were determined 6 hours following Aβ exposure. EPO, Wnt1, or combined EPO with Wnt1 administration significantly increased the expression of p-mTOR and p-p70S6K to a similar levels 6 hours following Aβ exposure. Yet, combined application of the PI 3-K inhibitor LY294002 (10 μM, given 1.5 hours prior to Aβ) with EPO, Wnt1, or combined EPO with Wnt1 resulted in a significant decrease in expression of p-mTOR and p-p70S6K (*P<0.01 vs. Aβ; ^†^*P*<0.01 vs. EPO/Aβ, Wnt1/Aβ, or EPO/Wnt1/Aβ). **(E** and **F)** EPO (10 ng/ml), Wnt1 (100 ng/ml), or combined EPO with Wnt1 were applied to microglial cultures 1 hour prior to Aβ exposure and cell survival was determined by the using trypan blue dye exclusion method 24 hours later. Representative pictures **(E)** and quantitative results (**F**) indicated that EPO, Wnt1, or combined EPO with Wnt1 application significantly reduced trypan blue staining and increased cell survival to a similar level following Aβ(10 μM) exposure. Application of the mTOR specific inhibitors rapamycin (Rapa, 50 nM) or KU 0063794 (KU, 100 nM) 1.5 hours prior to Aβ administration prevented EPO, Wnt1, or combined EPO with Wnt1 to foster cell survival and resulted in an increased staining of trypan blue and a decrease in cell survival in microglia (*P<0.01 vs. Aβ; †*P*<0.01 vs. EPO/Aβ, Wnt1/Aβ, or EPO/Wnt1/Aβ). Each data point represents the mean and SEM from 6 experiments.

The mammalian target of rapamycin (mTOR) and p70S6K are downstream targets of Akt1 [52, 53]. Since they are phosphorylated and activated through Akt1, we investigated whether EPO could alter mTOR and p70S6K activity during Aβ exposure. Western blot assay for phosphorylated mTOR (p-mTOR) (activated form of Akt1) and phosphorylated p70S6K (p-p70S6K) (activated form of p70S6K), a down stream target of mTOR, were performed following Aβ exposure. As shown in Figure 3A, the expression of p-mTOR and p-p70S6K was increased 6, 12, and 24 hours following Aβ exposure alone. In contrast, EPO (10 ng/ml) with a 1 hour pretreatment significantly increased and maintained the expression of p-mTOR and p-p70S6K at 6, 12, and 24 hours following Aβ exposure. As shown in Figure 3B, gene reduction of *Wnt1* with siRNA during EPO (10 ng/ml) application resulted in significantly decreased expression of p-mTOR and p-p70S6K 6 hours following Aβ exposure, suggesting that EPO increases mTOR and p70S6K activity through Wnt1. In Figures 3C and 3D, application of the phosphoinositide 3-kinase (PI 3-K) inhibitor LY294002 (10 μM) during treatment with either EPO (10 ng/ml) or Wnt1 (100 ng/ml) prevented EPO or Wnt1 from phosphorylating mTOR and p70S6K, illustrating that the PI 3-K and Akt1 pathways are necessary for EPO or Wnt1 to phosphorylate mTOR and p70S6K. The inhibitor LY294002 (10 μM) reversibly competes for ATP binding with PI 3-K [54]. In addition, combined application of EPO (10 ng/ml) and Wnt1 (100 ng/ml) phosphorylated mTOR and p70S6K to the same level as either agent applied independently and phosphorylation of mTOR and p70S6K was lost during application of LY294002 (10 μM), suggesting that EPO and Wnt1 use a common pathway to activate mTOR and p70S6K involving PI 3-K and Akt1 (Figures 3C and 3D).

We assessed whether EPO and Wnt1 required the mTOR pathway to prevent microglial cell injury. Microglial cell injury was determined by the trypan blue dye exclusion method 24 hours following Aβ exposure. As shown in Figures 3E and 3F, Aβ exposure results in a significant increase in trypan blue staining in microglia. EPO (10 ng/ml) or Wnt1 (100 ng/ml) applied 1 hour prior to Aβ exposure significantly reduced trypan blue staining in microglia (Figures 3E and 3F). Yet, administration of the mTOR inhibitor rapamycin (RAPA, 50 nM) or the specific mTOR inhibitor Ku 0063794 (KU, 100 nM) [55] with EPO (10 ng/ml), Wnt1 (100 ng/ml), the combination of EPO (10 ng/ml) and Wnt1 (100 ng/ml) blocked the ability of EPO and Wnt1 to prevent microglial cell injury during Aβ exposure, suggesting the mTOR pathway was necessary for EPO and Wnt1 microglial protection (Figures 3E and 3F).

### EPO phosphorylates Bad and fosters mitochondrial organelle release of Bad through Wnt1 and mTOR

**Figure 4:**
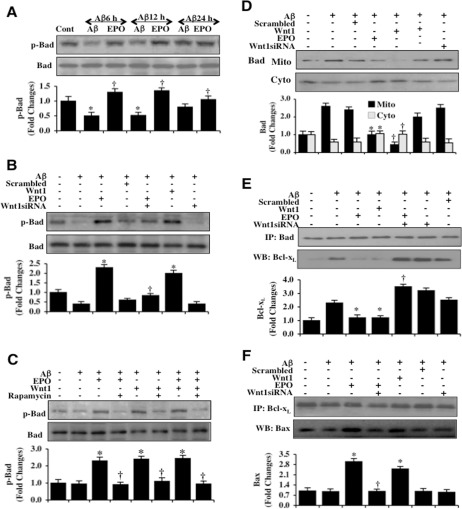
EPO through Wnt1 phosphorylates Bad, controls mitochondrial trafficking of Bad, and modulates Bad, Bcl-xL, and Bax binding (**A**) Microglial protein extracts (50 μg/lane) were immunoblotted with phospho-rylated Bad (p-Bad, (Ser136)) antibody at 6, 12, and 24 hours following administration of Aβ (10 μM). Aβ exposure resulted in a significant decrease in the expression of p-Bad. EPO (10 ng/ml) with a 1 hour pretreatment significantly increased the expression of p-Bad 6 hours following Aβ exposure (*P <0.01 vs. Control; ^†^*P*<0.01 vs. Aβ of corresponding exposure time). In all cases, each data point represents the mean and SEM from 3 experiments. (**B**) Gene reduction of Wnt1 was performed with transfection of Wnt1 siRNA prior to Aβ (10 μM) administration in microglia. The expression of p-Bad was determined at 6 hours following Aβ exposure. EPO (10 ng/ml) or Wnt1 (100 ng/ml) with 1 hour pretreatments significantly increased the expression of p-Bad 6 hours following Aβ exposure. Yet, Wnt1 siRNA transfection prior to Aβ exposure prevented EPO from significantly phosphorylating Bad. Non-specific scrambled siRNA did not significantly alter p-Bad expression during Aβ exposure (*P < 0.01 vs. Aβ; ^†^P<0.01 vs. EPO/Aβ). (**C**) EPO (10 ng/ml), Wnt1 (100 ng/ml) or combined EPO with Wnt1 with 1 hour pretreatments significantly increased the expression of p-Bad 6 hours following Aβ (10 μM) exposure. Application of the mTOR inhibitor rapamycin (50 nM) with EPO (10 ng/ml), Wnt1 (100 ng/ml) or combined EPO with Wnt1 1.5 hour prior to Aβ exposure resulted in the loss of the ability of EPO, Wnt1, or combined EPO with Wnt1 to increase in the expression of p-Bad during Aβ exposure (*P < 0.01 vs. Aβ; ^†^*P*<0.01 vs. EPO/Aβ, Wnt1/Aβ or EPO/Wnt1/Aβ). (**D**) Gene reduction of Wnt1 was performed with transfection of Wnt1 siRNA prior to Aβ (10 μM) administration in microglia and the expression of Bad in both cytosolic and mitochondrial fractions was determined at 6 hours following Aβ exposure. EPO (10 ng/ml) or Wnt1 (100 ng/ml) with 1 hour pretreatments significantly reduced mitochondrial expression of Bad and increased the cytosolic expression of Bad following Aβ exposure. Yet, gene reduction of Wnt1 with Wnt1 siRNA transfection led to the loss of the ability of EPO to promote the release of Bad for the mitochondria to the cytosol during Aβ exposure. Non-specific scrambled siRNA did not significantly change the translocation of Bad during Aβ exposure (*P < 0.01 vs. Aβ; ^†^*P* < 0.01 vs. EPO/Aβ). (**E**) Gene reduction of Wnt1 was performed in microglia with transfection of Wnt1 siRNA prior to Aβ (10 μM) administration. Protein extracts were immunoprecipitated using Bad antibody 6 hours following Aβ exposure. Western blot for Bcl-xL expression in the precipitates was performed. EPO (10 ng/ml) or Wnt1 (100 ng/ml) with 1 hour pretreatments decreased binding of Bcl-xL to Bad during Aβ exposure. Gene reduction of Wnt1 with Wnt1 siRNA transfection prevented EPO to decrease the binding of Bcl-xL to Bad during Aβ exposure. Non-specific scrambled siRNA did not significantly alter the binding of Bad to Bcl-xL during Aβ exposure (*P < 0.01 vs. Aβ; ^†^*P*<0.01 vs. EPO/Aβ). (**F**) Gene reduction of Wnt1 was performed with transfection of Wnt1 siRNA prior to Aβ (10 μM) administration in microglia. Protein extracts were immunoprecipitated using Bcl-xL antibody 6 hours following Aβ exposure. Western blot for Bax expression in the precipitates was performed. EPO (10 ng/ml) or Wnt1 (100 ng/ml) with 1 hour pretreatments increased the binding of Bcl-xL to Bax during Aβ exposure. Gene reduction of Wnt1 with Wnt1 siRNA transfection prevented EPO to increase the binding of Bcl-xL to Bax during Aβ exposure. Non-specific scrambled siRNA did not significantly alter the binding of Bcl-xL with Bax during Aβ exposure (*P < 0.01 vs. Aβ; ^†^*P*<0.01 vs. EPO/Aβ).

Given that EPO utilizes Wnt1 and mTOR for microglial cytoprotection, we investigated the ability of EPO to phosphorylate Bad, an important modulator of cell injury and a downstream target of Akt1 at the phosphorylation site of Bad (Ser^136^) [43, 56]. As shown in Figure 4A, phosphorylation of Bad (p-Bad) was decreased at 6 hours and 12 hours following exposure to Aβ alone. In contrast, EPO (10 ng/ml) applied 1 hour prior to Aβ exposure significantly increased the expression of p-Bad at 6 hours, 12 hours, and 24 hours following Aβ exposure. Yet, the ability of EPO (10 ng/ml) to phosphorylate Bad 6 hours following Aβ exposure was lost with the gene reduction of *Wnt1*, suggesting that EPO requires Wnt1 to phosphorylate Bad and lead to its activation. Use of non-specific scrambled siRNA did not alter the phosphorylation of Bad during Aβ exposure. In addition, microglia treated with Wnt1 siRNA were also unable to phosphorylate Bad during Aβ exposure (Figure 4B). In Figure 4C, administration of the mTOR inhibitor rapamycin (50 nM) prevented both EPO (10 ng/ml) and Wnt1 (100 ng/ml) from phosphorylating Bad 6 hours following Aβ exposure, illustrating that EPO as well as Wnt1 required mTOR to phosphorylate Bad during Aβ exposure. In addition to maintaining the phosphorylation of Bad, EPO and Wnt1 also foster the translocation of Bad from mitochondria to the cytosol. In Figure 4D, EPO (10 ng/ml) and Wnt1 (100 ng/ml) independently promote the release of Bad into the cytosol from microglial mitochondria 6 hours after Aβ exposure. Loss of Wnt1 during gene reduction of *Wnt1* abrogates the ability of EPO to lead to the release of Bad from mitochondria to the cytosol. Use of non-specific scrambled siRNA did not alter the phosphorylation of Bad during Aβ exposure (Figure 4D).

### EPO and Wnt1 release Bad from Bcl-x_L_ and increase the binding of Bcl-x_L_ to Bax

We subsequently examined the ability of EPO and Wnt1 to alter Bad and Bcl-x_L_ binding as well as the association between Bcl-x_L_ and Bax. EPO (10 ng/ml) or Wnt1 (100 ng/ml) were applied 1 hour prior to Aβ exposure and microglial cell extracts at 6 hours following Aβ were immunoprecipitated using anti-bodies to Bad, Bcl-x_L_, and Bax. As shown in Figure 4E, representative western blots for Bad and Bcl-x_L_ immunoprecipitation demonstrates that Aβ exposure alone as well as with non-specific scrambled siRNA resulted in a significant increase in the expression of the Bad/Bcl-x_L_ complex when compared to untreated controls. In contrast, EPO (10 ng/ml) and Wnt1 (100 ng/ml) significantly reduced the expression of the Bad/Bcl-x_L_ complex when compared with untreated control cultures (Figure 4E). Furthermore, gene reduction of *Wnt1* during EPO treatment with Aβ results in significant expression of the Bad/Bcl-x_L_ complex, suggesting that Wnt1 is vital to allow Bad to release Bcl-x_L_ to block apoptosis. In Figure 4F, Aβ exposure alone as well as the non-specific scrambled siRNA result in a significant decrease in the expression of the Bcl-x_L_/Bax complex when compared to untreated microglial controls. Yet, EPO (10 ng/ml) and Wnt1 (100 ng/ml) significantly increase the expression of the Bcl-x_L_/Bax complex during Aβ exposure. The ability of EPO to block the apoptotic cascade by maintaining the Bcl-x_L_/Bax complex [57] is lost during *Wnt1* gene reduction, illustrating that Wnt1 is also critical to fostering the Bcl-x_L_/Bax complex during Aβ exposure.

### EPO prevents mitochondrial membrane depolarization during Aβ exposure through Wnt1

**Figure 5:**
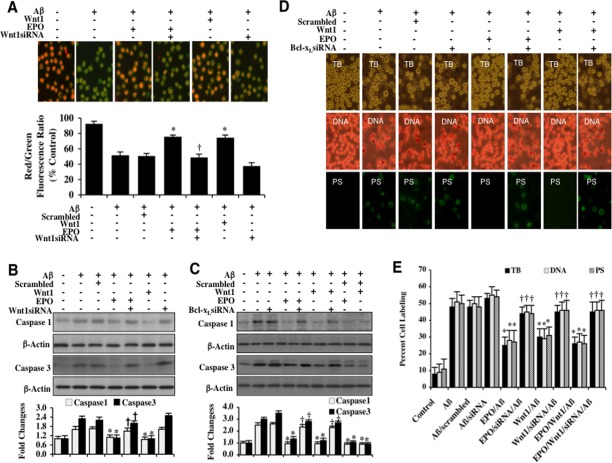
EPO and Wnt1 control mitochondrial membrane potential, block early and late apoptotic microglial Aβdegeneration, and prevent caspase 1 and 3 activation through Bcl-x_L_ **(A)** Representative images and quantitative results from JC-1 staining illustrate that Aβ (10 μM)results in a significant decrease in the red/green fluorescence intensity ratio of mitochondria within 6 hours when compared with untreated control cultures, demonstrating that Aβ exposure leads to significant mitochondrial membrane depolarization. EPO (10 ng/ml) or Wnt1 (100 ng/ml) with 1 hour pretreatments significantly increase the red/green fluorescence intensity of mitochondria in microglia, demonstrating that mitochondrial membrane potential was restored. In contrast, gene reduction of *Wnt1* with transfection of Wnt1 siRNA increased mitochondrial membrane depolarization to a greater degree than Aβ exposure alone and prevented the ability of EPO to maintain mitochondrial membrane potential during Aβ exposure. The relative ratio of red/green fluorescent intensity of mitochondrial staining was measured in 6 independent experiments with analysis performed using the public domain NIH Image program (*http://rsb.info.nih.gov/nih-image*) (*P<0.01 *vs*. Aβ; †*P* <0.01 *vs*. EPO/Aβ). **(B)** Microglial cell protein extracts (50 μg/lane) were immunoblotted with cleaved caspase 1 (active) and cleaved caspase 3 (active) antibodies 6 hours following Aβ(10 μM) exposure.Aβ(10 μM) exposure significantly increased caspase 1 and caspase 3 activities. In contrast, EPO (10 ng/ml) or Wnt1 (100 ng/ml) administration significantly decreased the expression of cleaved (active) caspase 1 and caspase 3 at 6 hours following Aβ(10 μM) exposure. Gene reduction of *Wnt1* with transfection with Wnt1 siRNA abrogated the ability of EPO to prevent caspase activation (*P<0.01 vs. Aβ; †*P* <0.01 vs. EPO/Aβ). Non-specific scrambled siRNA did not significantly change the expression of cleaved caspase 1 and caspase 3 during Aβ exposure. Each data point represents the mean and SEM from 3 experiments. Quantification of western band intensity from 3 experiments was performed using the public domain NIH Image program (*http://rsb.info.nih.gov/nih-image*). **(C)** Microglial cell protein extracts (50 μg/lane) were immunoblotted with cleaved caspase 1 (active) and cleaved caspase 3 (active) antibodies 6 hours following Aβ(10 μM) exposure.Aβ(10 μM) exposure significantly increased caspase 1 and caspase 3 activities. In contrast, EPO (10 ng/ml) or Wnt1 (100 ng/ml) administration significantly decreased the expression of cleaved (active) caspase 1 and caspase 3 at 6 hours following Aβ(10 μM) exposure. Gene reduction of *Bcl-x_L_*with transfection with Bcl-x_L_ siRNA abrogated the ability of EPO to prevent caspase activation (*P<0.01 vs. Aβ; †*P* <0.01 vs. EPO/Aβ). Non-specific scrambled siRNA did not significantly change the expression of cleaved caspase 1 and caspase 3 during Aβ exposure. Each data point represents the mean and SEM from 3 experiments. Quantification of western band intensity from 6 experiments was performed using the public domain NIH Image program (*http://rsb.info.nih.gov/nih-image*). **(D** and **E)**EPO (10 ng/ml) or Wnt1 (100 ng/ml) were administered to microglial cultures 1 hour prior to the Aβ (10 μM) exposure and trypan blue dye exclusion, DNA fragmentation, and membrane PS exposure were determined 24 hours later.Representative images **(B)** and quantitative analysis **(C)** demonstrate that Aβ(10 μM) results in a significant increase in trypan blue staining, DNA fragmentation, and membrane PS exposure in microglia 24 hours after Aβ exposure compared to untreated control cultures. In contrast, EPO (10 ng/ml), Wnt1 (100 ng/ml), or combined EPO and Wnt1 applied 1 hour prior to Aβ significantly reducedtrypan blue staining, DNA fragmentation, and membrane PS exposure in microglia 24 hours after Aβ exposure. Gene reduction of *Bcl-x_L_*with transfection of Bcl-x_L_ siRNA prior to Aβ exposure prevented EPO (10 ng/ml), Wnt1 (100 ng/ml), or combined EPO and Wnt1 from blocking cell injury and resulted in increased trypan blue staining, DNA fragmentation, and membrane PS exposure in microglia 24 hours following Aβ exposure. Non-specific scrambled siRNA did not significantly change cell injury during Aβ exposure (*P < 0.01 vs. Aβ; ^†^*P* <0.01 *vs*. EPO/Aβ, Wnt1/Aβ, or EPO/Wnt1/Aβ). Each data point represents the mean and SEM from 6 experiments.

In Figure 5A, Aβ exposure yielded a significant decrease in the microglial mitochondrial red/green fluorescence intensity ratio at 6 hours following Aβ exposure when compared to untreated control microglia, suggesting that Aβ exposure results in mitochondrial membrane depolarization. EPO (10 ng/ml) or Wnt1 (100 ng/ml) administration 1 hour prior to Aβ exposure significantly increased the red/green fluorescence intensity of the mitochondria, illustrating that EPO or Wnt1 can maintain mitochondrial permeability transition pore membrane potential during Aβ exposure. In contrast, EPO (10 ng/ml) could not maintain mitochondrial membrane potential during gene reduction of *Wnt1*, illustrating that Wnt1 modulates mitochondrial membrane potential for EPO (Figure 5A). Non-specific scrambled siRNA did not alter mitochondrial membrane potential during Aβ exposure.

### EPO and Wnt1 require Bcl-x_L_ to prevent caspase 1 and caspase 3 activation during Aβ exposure

We subsequently examined the ability of EPO and Wnt1 to control caspase 1 and caspase 3 activities. In Figures 5B and 5C, the expression of cleaved (active) caspase 1 and caspase 3 on western analysis were significantly increased at 6 hours following Aβexposure. EPO (10 ng/ml) or Wnt1 (100 ng/ml) application significantly blocked caspase 1 and caspase 3 activity during Aβexposure (Figures 5B and 5C). In contrast, transfection with Wnt1 siRNA during EPO treatment resulted in a significant elevation in caspase 1 and caspase 3 activities, suggesting that Wnt1 in microglial mediates the ability of EPO to control caspase activity during Aβexposure (Figure 5B). In addition, transfection with Bcl-x_L_ siRNA during EPO (10 ng/ml) or Wnt1 (100 ng/ml) treatment blocked the ability of EPO or Wnt1 to control caspase 1 or caspase 3 activity, illustrating that Bcl-x_L_ ultimately governs the ability of EPO and Wnt1 to regulate caspase 1 and caspase 3 activity in microglia during Aβexposure (Figure 5C). Non-specific scrambled siRNA did not alter the ability of EPO or Wnt1 to suppress caspase 1 or caspase 3 activity, supporting the specificity of Bcl-x_L_ through EPO or Wnt1 to regulate these pathways (Figure 5C).

### EPO and Wnt1 block early and late apoptotic microglial degeneration through Bcl-x_L_ during Aβ exposure

In Figures 5D and 5E, microglial cell injury was assessed with trypan blue staining. Early apoptotic injury was assessed by membrane PS exposure (annexin V staining) and late apoptotic genomic DNA fragmentation was assessed by TUNEL 24 hours following Aβ exposure. Treatment with EPO (10 ng/ml) or Wnt1 (100 ng/ml) blocked cell injury, early apoptotic injury with membrane PS exposure, and late genomic DNA fragmentation (Figures 5D and 5E). However, gene reduction of *Bcl-x_L_* with siRNA during EPO or Wnt1 application significantly prevented protection by EPO or Wnt1, illustrating that Bcl-x_L_ is necessary for EPO and Wnt1 prevention of microglial degeneration during Aβ exposure (Figures 5D and 5E). Combined treatment with EPO (10 ng/ml) and Wnt1 (100 ng/ml) also resulted in similar protection when compared with EPO or Wnt1 alone but combined protection was lost during gene reduction of *Bcl-x_L_* with siRNA, suggesting that EPO and Wnt1 rely upon Bcl-x_L_ in microglia to foster protection against Aβ (Figures 5D and 5E). Non-specific scrambled siRNA did not alter microglial survival or apoptotic injury during Aβ exposure.

## DISCUSSION

Therapeutic strategies that target the accumulation and toxicity of Aβ in the brain during Alzheimer's disease may offer significant promise for the treatment of this neurodegenerative disorder [2, 5, 58]. In particular, focus upon central nervous system microglia, immune cell sentinels that can sequester Aβ[4, 9, 14-16], may offer great promise for new therapies. Furthermore, identification of microglial cytoprotective pathways for entities such as EPO and Wnt1 [59-61] may synergistically enhance the development of treatments for Alzheimer's disease.

We show that EPO protects microglia against Aβ exposure during both early and late phases of apoptotic cell injury similar to prior studies with other injury models in non-neuronal cells [33, 34, 62]. During early apoptotic injury, externalization of membrane PS residues lead to the phagocytic removal of functional cells such as erythrocytes during anemia [63], neurons during oxidative stress [3, 64], and vascular cells during metabolic disorders and hypercoagulable states [51, 65]. As a result, EPO blocks early apoptotic signaling in microglia and may assist with tissue repair, regeneration, or the removal of cancer cells by maintaining the presence of functional microglia [16, 33, 66]. EPO maintains microglia during Aβ exposure through Wnt1 consistent with prior studies with wingless signaling pathways [20, 34, 45], since gene reduction of *Wnt1* prevents the onset of early and late apoptotic injury in microglia by EPO. However, we now illustrate that Wnt1 employs pathways that extend beyond traditional canonical and non-canonical Wnt signaling [36, 67-70]. We demonstrate that EPO through Wnt1 controls PI 3-K/Akt1 signaling to promote microglial survival during Aβ exposure, consistent with the known cytoprotective role for PI 3-K and Akt1. Wnt1 has previously been shown to activate the PI 3-K/Akt1 pathway during serum deprivation, ischemic injury, experimental diabetes [4, 20, 41, 43, 48] as well as tumor growth [71, 72]. Akt can foster cell growth and survival during inflammation, cardiovascular disease, and neurodegeneration [20, 21, 37, 50, 73, 74].

Once Akt1 becomes active through PI 3-K signaling, mTOR and p70S6K are also phosphorylated and activated [53, 75]. Wnt1 has been shown to require mTOR activation during breast cancer cell proliferation [76], to drive hair follicle proliferation and stem cell modulation [77], and to promote inflammatory cell survival during oxidant stress [34]. We now show that microglial survival during Aβ toxicity is dependent upon the activation of mTOR and p70S6K through both EPO and Wnt1 following PI 3-K and Akt1 activation. Combined treatment with EPO and Wnt1 did not provide a synergistic increase in the levels of phosphorylation of mTOR and p70S6K, but inhibition of the PI 3-K/Akt1 pathway prevented EPO and Wnt1 from activating mTOR and p70S6K, suggesting that Wnt1 may be the common pathway for EPO activation of mTOR and p70S6K.

EPO can block apoptotic injury through the maintenance of mitochondrial membrane potential and the inhibition of caspase activity during cardiovascular injury, renal disease, metabolic injury, and neurodegeneration [20, 22, 23, 28, 78-80]. Wnt signaling also has been shown to modulate mitochondrial and caspase apoptotic pathways in a number of cell types [4, 43, 71]. Common to both EPO and Wnt1 through our present work, PI 3-K and Akt1 also can oversee mitochondrial membrane permeability and caspase activation [49-51]. We now demonstrate in a series of studies that Wnt1 maintains mitochondrial membrane permeability and blocks caspase activation through Bad, Bax, and Bcl-x_L_. First, EPO and Wnt1 during Aβ exposure phosphorylate Bad and promote the translocation of Bad from the mitochondria to the cytosol of microglia. Phosphorylation of Bad is controlled by Wnt1 [43], but in addition we show that Bad phosphorylation through Wnt1 also requires the activation of mTOR which is necessary for microglial survival [75]. A pro-apoptotic Bcl-2 family member, Bad can be phosphorylated through Akt, bind to the cytosolic protein 14-3-3 to release Bcl-x_L_, and allow Bcl-x_L_ to bind to the pro-apoptotic protein Bax [24, 81-83]. Bcl-x_L_ is necessary to block Bax translocation to the mitochondria, maintain mitochondrial membrane potential, and prevent the release of cytochrome c from the mitochondria [39, 59]. Second, we demonstrate that EPO and Wnt1 significantly limit the expression of the Bad/Bcl-x_L_ complex during Aβ exposure and that Wnt1 is critical to allow Bad to release Bcl-x_L_ to block apoptosis. Furthermore, we show that Wnt1 is necessary for EPO to promote the Bcl-x_L_/Bax complex during Aβ exposure to prevent Bax translocation to the mitochondria.

We illustrate that EPO through Wnt1 also maintains microglial mitochondrial membrane permeability during Aβ exposure. As a result, the activity of caspase 1 and caspase 3, mediators of early and late phases of the apoptotic cascade [47, 84-86], are also blocked through Wnt1. EPO has been shown to rely upon modulation of Bcl-x_L_ levels to promote cellular protection [22, 87, 88]. In addition, Akt1 has been shown to prevent early PS membrane mediated apoptotic injury [22, 89] that is regulated through Bcl-x_L_ expression [22, 50, 90, 91]. We now show that EPO controls early and late apoptotic injury and caspase 1 and caspase 3 activities in microglia during Aβexposure through Wnt1 that oversees Bcl-x_L_ expression.

Our studies identify novel signal transduction pathways of Aβ degeneration for the protection of central nervous system microglia. In this respect, EPO through Wnt1 governs early and late apoptotic Aβ microglial injury through non-traditional canonical and non-canonical pathways that involve the integration of the PI 3-K/Akt1 pathways, mTOR, and mitochondrial related signaling of Bad, Bax, and Bcl-x_L_.Future studies that further elucidate the pathways of cell toxicity for microglia during Aβ exposure may open new approaches for previously unrecognized strategies against Alzheimer's disease and other degenerative disorders.

## MATERIALS AND METHODS

### Microglial cell cultures

Per our prior protocols, the microglial cell line EOC 2 was obtained from American Type Culture Collection (ATTC, Manassas, VA.) [34, 43]. Cells were maintained in Dulbecco's modified Eagle medium (ATTC, Manassas, VA), supplemented with 10% heat-inactivated fetal bovine serum (Sigma, St Louis, MO), 50 μg/ml penicillin and streptomycin and 20% media from the LADMAC cell line (ATCC, Manassas, VA) which contains colony stimulating factor-1 (CSF-1) secreted by LADMAC cells. Cells were seeded onto 24-well plates or 35 mm culture dishes at a density of 1.5 × 10^6^ cells per well or 4 × 10^6^ cells per dish.

### Experimental treatments

Per our prior protocols [3, 4, 34], β-amyloid (Aβ_1-42_) (Invitrogen, Carlsbad, CA) was dissolved in PBS at a concentration of 100 μM. To allow for Aβ aggregation, Aβ was incubated at 37°C for a 7 day period and then directly applied to microglial cell cultures per the experimental protocols. For treatments applied prior to Aβ, human recombinant erythropoietin (EPO) (10 ng/ ml, Sigma, St Louis, MO), human recombinant Wnt1 protein (100 ng/ml, R&D Systems, Minneapolis, MN), the mammalian target of rapamycin (mTOR) inhibitors rapamycin (RAPA, 20 nM, Tocris, Ellisville, MO) or Ku 0063794 (KU, 100 nM, Tocris, Ellisville, MO) were continuous. For PI 3-K inhibition, LY294002 (Calbiochem, La Jolla, CA) was added directly to the cultures 1 hour prior to Aβ application and the treatment of PI 3-K inhibition was continuous.

### Assessment of cell survival

Microglial injury was determined by bright field microscopy using a 0.4% trypan blue dye exclusion method 24 hours following treatment with Aβ per our previous protocols [34, 43]. The mean survival was determined by counting eight randomly selected non-overlapping fields with each containing approximately 10-20 cells (viable + non-viable). Each experiment was replicated 6 times independently with different cultures.

### Assessment of DNA fragmentation

Genomic DNA fragmentation was determined by the terminal deoxynucleotidyl transferase nick end labeling (TUNEL) assay [24, 50]. Briefly, microglial cells were fixed in 4% paraformaldehyde/0.2% picric acid/0.05% glutaraldehyde and the 3'-hydroxy ends of cut DNA were labeled with biotinylated dUTP using the enzyme terminal deoxytransferase (Promega, Madison, WI) followed by streptavidin-peroxidase and visualized with 3,3'-diaminobenzidine (Vector Laboratories, Burlin-game, CA).

### Assessment of membrane phosphatidylserine (PS) membrane externalization

Externalization of membrane PS residues was determined by using Annexin V labeling per our prior studies [24, 50]. A 30 μg/ml stock solution of Annexin V conjugated to phycoerythrin (PE) (R&D Systems, Minneapolis, MN) was diluted to 3 μg/ml in warmed calcium containing binding buffer (10 mmol/L Hepes, pH 7.5, 150 mmol/L NaCl, 5 mmol/L KCl, 1 mmol/L MgCl_2_, 1.8 mmol/L CaCl_2_). Plates were incubated with 500 μl of diluted Annexin V for 10 minutes. Images were acquired with “blinded” assessment with a Leitz DMIRB microscope (Leica, McHenry, IL) and a Fuji/Nikon Super CCD (6.1 megapixels) using transmitted light and fluorescent single excitation light at 490 nm and detected emission at 585 nm.

### Gene reduction of Wnt1 and Bcl-x_L_ with siRNA transfection

Microglia were plated into 35 mm dishes or 24 well plates. To silence *Wnt1* gene expression, Wnt1 siRNA was selected targeting the mouse Wnt1 mRNA. This siRNA consists of a RNA duplex containing a sense strand 5'-GCAGUACAACAUCGAUUUtt-3' and an antisense strand 5'-AAAUCGAUGUUGUCACUGCag-3'. The siRNA was designed by using *Silencer*® siRNA construction kit synthesized by Ambion (Austin, TX). To silence *Bcl-x_L_* gene expression, commercial reagents using the SMARTpool siRNA pool for Bcl-x_L_(Santa Crutz, Santa Crutz, CA) were used. Transfection of siRNA duplexes were performed with Lipofectamine 2000 reagent according to manufacturer guidelines (Invitrogen, Carlsbad, CA). Experimental assays were performed 72 hours post-transfection. For each siRNA assay, negative controls contain multiple siRNAs including the target siRNA and positive controls are absent of the target siRNA.

### Expression of phosphorylated Akt1, total Akt1, phosphorylated mTOR, total mTOR, phosphorylated p70S6K, total p70S6K, Wnt1, Bad, Bcl-x_L_, and Bax

Cells were homogenized and each sample (50 μg/lane) was subjected to SDS-polyacrylamide gel electrophoresis (7.5% for Akt, mTOR, p70S6K; 12.5% for Wnt1, Bad, Bcl-x_L_, and Bax). After transfer, the membranes were incubated with a rabbit polyclonal antibody against Wnt1 (1:1000, R&D Systems, Minneapolis, MN), a rabbit monoclonal antibody against phospho-Akt1 (Ser^473^, 1:1000) and total Akt1 (1:1000) (Cell Signaling, Beverly, MA), a rabbit monoclonal antibody against phospho-mTOR (Ser^2448^, 1:1000) and total mTOR (Cell Signaling, Beverly, MA), a rabbit antibody against phopho-p70S6K (Thr^389^, 1:1000) and total p70S6K (1:1000) (Cell signaling Technology, Beverly, MA), a primary rabbit against phospho-Bad (Ser^136^, 1:1000) (Cell signaling Technology, Beverly, MA), a primary rabbit against phospho-Bcl-x_L_ (1:1000) (Cell signaling Technology, Beverly, MA), or a primary rabbit against phospho-Bax (1:1000) (Cell signaling Technology, Beverly, MA). Following washing, the membranes were incubated with a horseradish peroxidase (HRP) conjugated secondary antibody goat anti-rabbit IgG (1:5000, Zymed Labora-tories, Carlsbad, CA). The antibody-reactive bands were revealed by chemiluminescence (Amersham Pharmacia Biotech, Piscataway, NJ) and band density was performed using the public domain NIH Image program (developed at the U.S. National Institutes of Health and available at http://rsb.info.nih.gov/nih-image/).

### Akt kinase activity assessment

Per our prior work [4, 50], Akt1 activity was determined by using a commercially available nonradioactive Akt1 kinase assay kit with a GSK-3β fusion protein. Cells were lysed in ice with 150 μl of lysis buffer containing 1% Triton X-100, 10% glycerol, 137 mM NaCl, 20 mM Tris-HCl (pH 7.5), 2 μg/ml aprotinin, 2 μg/ml leupeptin, 1 mM phenylmethylsulfonyl fluoride, 20 mM NaF, 1 mM Na_2_PPi, and 1 mM Na_3_VO4. Equal amounts of lysates (200 μg) were pre-cleared by centrifugation and pre-absorbed with protein A-protein G (1:1) agarose slurry. Immunoprecipitation was carried out over night using the immobilized anti-Akt1G1 monoclonal antibody (Cell Signaling Technology, Beverly, MA) cross-linked to agarose. Immunoprecipitates were washed three times with lysis buffer and twice with Akt kinase buffer (20 mM HEPES, pH 7.4, 10 mM MgCl_2_, 10 mM MnCl_2_). Kinase assays were performed for 30 min at 30°C under continuous agitation in kinase buffer containing 200 μM ATP and 1 μg of GSK-3 fusion protein according to the manufacturer's instructions (Cell Signaling Technology, Beverly, MA). Samples were analyzed by Western blot analysis using 12.5% SDS-polyacrylamide gel and rabbit antibody against p-GSK-3α/β (Cell Signaling Technology, Beverly, MA). Data for the kinase activity were expressed as percentage of control activity.

### Assessment of mitochondrial membrane potential

The fluorescent probe JC-1 (Molecular Probes, Eugene, OR), a cationic membrane potential indicator, was used to assess the mitochondrial membrane potential [21, 50]. Microglia in 35 mm dishes were incubated with 2 μg/ml JC-1 in growth mediumat 37 °C for 30 min. The cultures were washed three times using fresh growth medium. Mitochondria were then analyzed immediately under a Leitz DMIRB microscope (Leica, McHenry, IL, USA) with a dual emission fluorescence filter with 515-545 nm for green fluorescence and emission at 585-615 nm for red fluorescence.

### Immunoprecipitation of Bad, Bcl-x_L_ or Bax

Cell lysates of total protein (200 μg) were incubated with antibody against protein Bad (1:1000, Cell Signaling, Beverly, MA) or Bcl-x_L_ (1:1000, Cell Signaling, Beverly, MA) overnight at 4°C. The complexes were collected with protein A/G-agarose beads, centrifuged and then prepared for Bad, Bax, and Bcl-x_L_ western analysis.

### Statistical analysis

For each experiment, the mean and standard error were determined. Statistical differences between groups were assessed by means of analysis of variance (ANOVA) from 6 replicate experiments with the post-hoc Dunnett's test. Statistical significance was considered at P<0.05.
